# The Incident Management Measurement Tool (IMMT): A Tool for Measuring Public Health Incident Management During and After Emergencies

**DOI:** 10.1017/dmp.2025.10060

**Published:** 2025-06-16

**Authors:** Vanessa Parks, Aaron Clark-Ginsberg, Jalal Awan, Jay Balagna, Grace Hindmarch, Laura Fraade-Blanar, Holly Fisher, Sara Vagi, Paul Renard, Christopher D. Nelson

**Affiliations:** 1 RAND Corporation, Pittsburgh, Pittsburgh, PA, USA; 2 RAND Corporation, Santa Monica, CA, USA; 3 Pardee RAND Graduate School, Santa Monica, CA, USA; 4 Centers for Disease Control and Prevention, Atlanta, GA, USA

**Keywords:** incident management, public health emergency preparedness, measurement, emergency response, after action review, intra-action review, COVID-19

## Abstract

**Objectives:**

Risks and priorities change during the management of public health incidents. Here we describe a new tool, the Incident Management Measurement Tool (IMMT), that can be used to inform midcourse corrections during public health emergencies and realistic exercises.

**Methods:**

We developed the IMMT through a literature review and subject matter expert interviews. We field tested the tool in 23 incidents ranging in size, duration, and complexity, making changes based on user feedback.

**Results:**

The IMMT consists of 2 modular data collection methods, a survey of the incident management team and a protocol for a peer assessor. Pilot testing suggested that the tool is valid, reliable, feasible, and useful.

**Conclusions:**

Measurement of public health incident management is feasible and may be useful for improving response times and outcomes. Moreover, a limited set of standard measures is relevant to a wide range of incident response contexts.

Public health incident management is the structured process by which responders coordinate and make adaptive decisions to direct resources and capabilities effectively, addressing and mitigating evolving health risks in affected communities. As a critical part of public health,^1^ effective incident management involves answering questions about who is in charge, what resources are available, how the threat is evolving, and how to pay for the response.^2^

Risks and priorities change during responses, such as the shift from disease containment to mitigation during a disease outbreak. Thus, incident managers must be ready to update plans, reconfigure staff and resources, reexamine key assumptions, coordinate activities, and effectively communicate that information – often in short order.^3^ Such adjustments could be aided by feasible, useful, accurate tools for assessing and improving incident management during and after incidents.

To date there have been some important steps toward improving measurement and evaluation during incidents, starting with Chamberlain et al.’s proof-of-concept protocol for “intra-action reviews” (IAR) in the context of the Ebola response.^4^ Subsequently, the World Health Organization created a protocol for IARs for use by national health ministries and their partners during COVID-19, leveraging information from desk reviews of key documents and a group discussion with 10–20 participants.^5^ Similarly, the European Centre for Disease Prevention and Control created a 1-day COVID-19 IAR designed for use at national or subnational levels.^6^ These efforts were designed to support the management of public health incidents on a large scale.

Yet, there remains an unmet need for measurement tools that provide a more in-depth focus on the intra and after-action evaluation of public health incident management,^1,7^ particularly in cases with limited time and resources, such as small- and medium-sized health departments that often lack adequate evaluation resources. To best meet these needs, tools supporting public health incident management at smaller scales should be applicable across a wide range of public health incident management structures, which can range from top-down hierarchical structures to more collaborative networked approaches, and structures that incorporate aspects of policy (e.g., the U.S. National Incident Management System) selectively and variably over time.^8,9^

## Purpose

Here we present a new tool for health departments and their partners to improve public health incident management, the Incident Management Measurement Tool (IMMT). The IMMT is explicitly designed to measure key domains of incident management during and after public health incidents and realistic exercises, complementing other post-incident tools and approaches such as Homeland Security Exercise Evaluation Program^10^ and after-action reviews. The tool is designed to reduce the burden and improve the reliability of data collection and analysis in a way that meets the needs especially of medium- and small-sized communities, which often lack robust evaluation resources, and can be used across a variety of incident management structures.

We provide an overview of our field-testing process before describing the tool, explaining strengths and weaknesses identified from field testing, and concluding with a discussion of implications for research and practice.

## Materials and Methods

Development and testing of the tool was guided by 4 criteria commonly used in health care quality research^11^ and public health emergency preparedness.^12^ *Validity* is the extent to which indicators reflect key public health incident management constructs, and *reliability* is whether indicators produce similar results under consistent conditions and at different time points. While these criteria tend to be of interest to those focused on measurement, valid and reliable measures are of little use if people cannot use them. Thus, we seek to assess their *feasibility* and their *utility*, or the extent to which public health incident managers and partners can use insights from the findings to identify implications for action.

Development of the IMMT took place from 2018–2020 and pilot testing from 2020–2023, with close collaboration with the end users of our tool, public health incident managers.

### IMMT Initial Development Process (2018–2020)

Development of the IMMT was guided by the Public Health Emergency Incident Management System conceptual framework previously published in this journal,^13^ which drew upon incident management-related research, theory, and doctrine, as well as 50 expert discussions. The framework conceptualizes incident management as comprised of 5 domains: situational awareness and information sharing, incident action and implementation planning, resource management and mobilization coordination and collaboration, and feedback and continuous quality improvement.

The process of identifying specific items for measurement in the tool began with 135 candidate items, which we refined to a set of 58 items through semi-structured interviews and numerical rankings by ~50 subject matter experts along our 4 criteria (validity, reliability, feasibility, and utility). Additional refinement during pilot testing reduced the total to 45 items, collected via 2 protocols: an incident management team survey and a peer assessment protocol. The protocols are modular (i.e., they can be used separately or in combination) and aligned with the 5 domains from the conceptual framework. The survey is intended to capture reflections from a broader range of public health incident management team members than would typically be possible using group discussion methods. Moreover, the use of fixed choice, Likert-scale items affords a degree of standardization and comparability in responses not provided by more discussion-oriented approaches, though this is supplemented by a number of free text response items, which allow respondents to elaborate on their responses and provide additional nuance. Seeking to minimize data collection burden, we aimed for the survey to take no more than 15 minutes for each respondent.

The peer assessment protocol focuses on aspects of public health incident management that involve judgment and consideration of context. For instance, 1 item asks whether “Situation assessments are delivered to the appropriate stakeholders in a timely manner,” recognizing that judging timeliness may involve considering incident tempo, hazard type, organization structure, and other contextual factors – and that timely and accurate information is critical in public health decision making. The peer protocol asks assessors to provide responses in addition to Likert-scale style items to nudge them to take a clear position. Similar to the survey, additional value comes through textual explanations provided with the multiple-choice responses. Selecting appropriate peer assessors, therefore, is critical, and the tool provides guidelines for selecting individuals who have enough (a) distance to offer an objective viewpoint and (b) relevant expertise to be credible. The protocol also offers detailed guidance on finding opportunities to observe relevant constructs in situ, via discussions with key individuals, observation, and reviewing documents and other artefacts (e.g., resource tracking systems).

Other materials in the toolkit are designed to facilitate use of the data collection protocols: guidelines for the lead evaluators and peer assessors (who are involved in data collection, analysis and interpretation), a communication template to help explain the process to partners, and a form for capturing important incident background information useful for interpreting the results. The toolkit concludes with guidance for interpreting the results. This guidance addresses the analysis of both individual survey items and domain-level summary scores, as well as analysis of themes in comments from the peer assessor. It provides focusing questions and strategies for combining results from the survey and peer protocol into key findings. Providing guidance on using data to support corrective action plans is beyond the tool’s scope, but the toolkit does provide links to other relevant resources. All these materials were made available digitally and could be administered online or by printing and sharing the materials. Table 1 summarizes the sections of the toolkit, and more information is available online at https://www.rand.org/pubs/tools/TLA3196-1.html.

### IMMT Pilot Testing Process (2020–2023)

Pilot testing was used to refine the IMMT to further align it to our 4 criteria (validity, reliability, feasibility, utility). As mentioned above, pilot testing began in 2020 during the COVID-19 pandemic. Given health departments’ focus at that time, initial tests focused on the management of the pandemic. Later testing focused on a broader set of incidents, including floods, wildfires, and exercises for mass shooting responses.

Pilot testing was guided by research questions related to uptake, proper use of the tool, quality of the resulting data, and usefulness to end users (see Table 2). Uptake of the tool by incident managers is important; if uptake is minimal, the tool will have limited effect in improving public health incident management. To assess uptake, we analyzed data from the recruitment process, which involved leveraging our professional connections, working through professional associations, and advertising through blogs, commentaries, and speaking at conferences. We targeted mostly small and medium-sized health departments, as these types of agencies might best benefit from a low-burden evaluation process. Moreover, given the range of actors involved in public health incident management, particularly during the COVID-19 pandemic, we sought to include not just health departments but also emergency management agencies, health care coalitions, and other types of agencies involved in public health incident management.

Data sources used during pilot testing include data collected using the measurement instruments and data from post-pilot site debriefs. This includes analyses of the survey items, which we analyzed by assessing item frequency distributions, measures of central tendency, Cronbach’s alpha, item-rest correlations, and other methods described in more detail below (quantitative analysis did not include peer assessor data since only 1 score was provided per peer assessment). This also included analyses of write-in comments from the survey and peer assessments. In addition, we conducted debriefs with the lead evaluator after each test, which focused on their experience using the tool and the tool’s strengths, weakness, and areas for improvement, as well as their assessment of how well aligned the responses were to their experiences in the response or exercise. To analyze debrief and write-in data, we used a basic qualitative coding frame consisting of 3 primary codes, with subcodes noted in parentheses background (background to organization, background to incident, description of testing process); assessment along criteria (validity, reliability, feasibility, utility, and an overall “Gestalt” assessment); conclusions (potential changes, key takeaways, other notes).

We revised the tool based on the results of these analyses at 3 points during the testing process. The first set of revisions (“wave 1”) came after testing of an initial draft and involved extensive revisions to the peer protocol and modest revisions to the survey protocol. The second and third revisions (“wave 2” and “wave 3”) involved successively smaller revisions to the protocols, including (a) deleting 2 survey items that early testing revealed (through item-rest correlations, Cronbach’s alpha, and user feedback) to be unclear, (b) adding a question to the incident background form, (c) making minor clarifications to 4 survey items, and (d) clarifying user instructions. Due to the changes made to the survey over these waves, it is not always appropriate to make comparisons between them. We indicate in the text where this is the case.

Recruitment of pilot sites was significantly impacted by the COVID-19 pandemic, which left many health departments too stretched to consider participating. Over the course of the project, we directly reached out to over 145 potential pilot sites and several dozen individuals with connections to potential pilot sites, and we conducted outreach through email listservs, social media, conference presentations, and brown bag meeting series.

Table 3 summarizes the pilot sites, the waves of testing, agency type, the methods used, and the type of incident in our 3 waves of testing. A total of 18 sites participated and 3 sites engaged in 2 rounds of data collection (marked with asterisks). Participants included not only state and county health departments but also others engaged in public health response, including fire and emergency management agencies addressing the health consequences of wildfires, and health care coalitions conducting exercises with various partners. Sites were given the option to administer the survey or peer protocol separately or together, although most elected to use the survey only to minimize burden. Most pilot tests were conducted during and after real incidents, but a handful of tests involved full-scale exercises (Table 3). The latter were carefully screened in advance to ensure the exercise provided sufficient opportunity to observe public health incident management activities.

## Results

Table 4 displays the key findings, by research question and criteria. Regarding uptake of the tool (Research Question 1), we found that recruitment was a challenge. Given the timing of pilot testing, many health departments were focused on their responses to the COVID-19 pandemic. That said, it appears as though pilot sites were able to use the tool to generate useful data (Research Question 2). Overall, we found that the tool provided high quality data (Research Question 3), and pilot sites reported that they found the tool to be worthwhile and useful (Research Question 4).

### Uptake of the tool

As noted above, recruitment of pilot sites was difficult due to competing priorities associated with the COVID-19 pandemic, and these challenges continued throughout pilot testing as sites dealt with staff burnout, turnover, and other lingering consequences. Thus, it is difficult to judge how successful uptake might be during future events.

### Ability to use the tool properly to generate data

Time spent using the tool did not seem to be particularly burdensome to participants. On average, respondents took approximately 14 minutes to complete the survey, under our 15-minute target. In debriefs, lead evaluators also stated that using the tool was not too burdensome, taking approximately 4–6 hours in total to organize data collection and collect and interpret results. Limited uptake of the peer assessment tool makes it difficult to judge with confidence, but experience suggests that it is possible to administer it within the target time of 8–12 hours, though 1 site decided to expand the peer assessor’s participation, which took more time.

Missing data did not appear to be a significant issue. Throughout the testing process, the percentage of data that were missing did not exceed 7% (wave 1), 1% (wave 2), and 4% (wave 3), respectively. This suggests the survey was neither too difficult, confusing, time intensive, nor inapplicable to respondents’ experiences.

The pilot tests did, however, reveal sources of confusion, which were addressed through revisions. For instance, earlier pilot test debriefs revealed confusion about to whom the lead evaluators would send the survey. Based on discussions during debriefs, it appears the confusion was related to the fact that COVID-19 public health incident management often included greater participation than usual because of the very active role that governors’ and mayor’s offices played during the pandemic. For later pilots, we refined the guidance on this point, and lead evaluators voiced less confusion during debriefs. It is also possible that novel incident management structures became more routine as the nation transitioned out of the pandemic or that personnel became more familiar with them.

Similarly, some participants voiced that they wanted more guidance on how to clearly define the specific time period of the incident response covered by data collection. This feedback appeared to be in response to the unusual length of the COVID-19 public health emergency. While this was primarily observed during pilots related to the COVID-19 pandemic, this pointed to revisions that might be useful for other types of incidents. In subsequent versions of the tool, we more clearly directed lead evaluators to select a specific time period or aspect of the response, and to clearly state this in an email distributing the survey to their team. Accordingly, we received less feedback about this in later pilot tests.

### Ability to use tool to produce high quality data

We used 2 “Gestalt” items (A and B) to serve as comparisons for how well the survey captured incident management (Gestalt A) and how well the incident was managed (Gestalt B). Results of the survey and subsequent debriefs suggest that users thought the instrument captured incident management processes and activities well. Beginning in Wave 2, we examined whether the means of these items differed between sites.

Possible scores for an item range from 1 (strongly disagree)-5 (strongly agree). Average scores for Gestalt A (*Overall, I feel this survey asked questions necessary to understand the management of this incident*) were 3.7 and 4.0 in Waves 2 and 3, respectively, and the scores for sites ranged from 3.2 to 4.75. ANOVA results pooling Waves 2 and 3 sites together suggest that there were significant differences by sites. However, when Waves 2 and 3 are analyzed separately, the ANOVA results suggest there are no differences by sites. Given the higher means in Wave 3, this may suggest that respondents in later pilots viewed the survey as more applicable, after revisions to the survey had been made. Debrief results align with this analysis; sites where respondents reported higher scores for Gestalt A tended to view the incidents as having been managed well, and those with lower scores expressed that there were areas for improvement.

Another way to assess whether the survey questions capture intended information (how well the incident was managed) is to compare the responses to Gestalt B (*Overall, I feel this incident was managed well* ) to the responses for all the domains for each site. This can be viewed as a type of criterion validity check, with Gestalt B as the criterion, albeit imperfect.

Because the framework included 5 key domains of incident management, we conducted analyses on the items within those domains to explore whether they consistently measured the appropriate concepts. As demonstrated in Table 5, the domain scores are not widely different from Gestalt B, suggesting that survey responses are consistent with overall views of the incident. In our debriefs, lead evaluators expressed that average domain scores aligned with how the incident was managed and did not express concerns in variations between Gestalt B scores and survey scores.

Analysis of domain summary scores suggested that average scores may reliably capture those associated constructs. We used Cronbach’s alpha to assess the internal consistency of items within the 5 domains that comprise public health incident management, which our survey is designed to measure (see Table 6). In social sciences, acceptable values of alpha are ≥0.70.^14^ Given the multiple changes made during Wave 1, we exclude Wave 1 from these analyses and present alphas for Waves 2 and 3. Values exceeded 0.70 for all domains and waves with the exception of Domain 5, which could be explained by the domain only containing 3 items. Therefore, we recommend caution when interpreting the results from Domain 5.

Write-in comments also represent opportunities for sites to delve more deeply into their team’s perceptions of the response and devising possible corrective actions. Across all waves of the pilot testing process, the majority of respondents provided write-in comments on the survey (52% in Wave 1, 50% in Wave 2, and 78% in Wave 3). This suggests that respondents are not skipping through the survey quickly and find value in sharing their perceptions about the response. In early pilots, these write-in responses helped us identify when survey items were inapplicable to respondents. Throughout the testing process, they provided an opportunity for respondents to provide nuance and to express strong feelings about the management of the incident.

### Users deem tool useful and worthwhile

When asked in debriefs, lead evaluators voiced that they found the tool was worth the time and effort, particularly for filling a gap between a quick hotwash and more time-intensive after-action review. Several sites appreciated the value provided relative to the survey’s brevity, while also providing options for write-in comments. One lead evaluator noted, “[the survey] helps me think about what to focus on and gets creative juices flowing on what to do.” The fact that 3 sites conducted a second pilot test may also be evidence of perceived benefit.

However, some respondents noted that scores on some indicators reflect broader institutional issues that tend to be out of their control (e.g., some sites attributed low scores on items related to staff wellbeing to issues related to resource scarcity and underinvestment in public health given growing public health needs). Even where beyond sites’ control, it is possible that users could use results to approach those who are in a position to make changes.

## Discussion

We set out to develop a tool that state and local public health incident managers with modest evaluation resources could use for public health incident management-related improvement efforts during and after emergency incidents. We conducted pilot tests in 23 sites with different public health agency types responding to incidents of varying sizes, durations, and complexities. Uptake was limited by the burdens imposed by the COVID-19 pandemic. Yet, the pilot tests show that measurement during incidents is possible, even under difficult circumstances. Moreover, those who used the tool were able to do so with only modest effort, generating data that they believed captured essential elements of public health incident management, correlated reasonably well with overall “Gestalt” judgments of public health incident management quality, and clustered reasonably well into the 5 domains of our conceptual framework.^13^ Pilot findings also suggest that it is possible to capture the essential elements of public health incident management across a wide variety of incidents (e.g., COVID-19, wildfires, medical surge events) and contexts (e.g., state and local health departments, emergency management agencies, health care coalitions) with a relatively limited number of standard data elements.

Nonetheless, there are important limitations that must be acknowledged. First, limited uptake not only raises questions about how scalable the tool might ultimately be, but also if the amount of data from field testing is sufficient. Despite our efforts to recruit a wide range of test sites, those willing and able to participate in the pilot were likely more motivated and had greater capacity to engage in data collection and analysis than the typical health department. As such, we may be missing the perspectives of more under resourced health departments. Simply making a tool available without additional inducements or supports may not be sufficient. Attaching the measures to some sort of clear incentive – such as making their use a condition of funding may be 1 way to increase uptake in the long term. Short of that, it might be worth considering integrating the tool into technical assistance activities with states and locals. Yet, linking measurement and improvement tools too closely with incentives can reduce public health incident managers’ willingness to collect and use data earnestly, fearing potential consequences – real or perceived – of poor performance.^15^ Furthermore, stemming from the issue of limited uptake, we are unable to determine if the tool performs differently in long-term incidents (e.g., COVID-19) versus short-term incidents.

A second limitation is that the focus of the tool is limited to collecting, analyzing and interpreting data on public health incident management, but stops short of guiding IMMT users through the process of using insights from data to identify root causes and effective corrective actions for addressing them. For example, the write-in responses are useful, but if the data are collected in such a way where the respondents are not identifiable, the lead evaluator may have limited ability to follow-up on issued that were raised. So, the tool provides a way to screen for potential issues, but further or repeated evaluation may be necessary, as part of larger evaluation processes, including intra- and after-action reviews.

In the future, use of the tool in the context of learning collaboratives might provide a forum for sharing success stories and identifying common strategies for moving towards action.^16,17^ This might occur at the regional level with agencies in the same geographic area or might be based on issue or population (with agencies serving an urban constituency, tribal, etc.).

Finally, while our tool is designed for use within US contexts, public health incident management involves a similar set of processes and activities globally. The IMMT might be useful in these other locations, including other countries where CDC is already providing emergency management assistance and training. Public health agencies experiencing high levels of resource scarcity might find the tool particularly beneficial, as it could provide an off-the-shelf approach for measuring performance.

## Figures and Tables

**Table 1.** Elements in the IMMT Toolkit

Section	Description
Read Me First	Overview of the tool its use
Lead Evaluator Materials	Guidelines for Lead Evaluator Communication Templates Incident Background Information Template Incident Management Team Survey
Peer Assessment Materials	Guidelines for Peer Assessor Peer Assessor Protocol
Using the Data	Instructions for analyzing and using data collected using the IMMT
Methodology	Description of how the IMMT was developed

**Table 2.** Research questions guiding pilot testing

	Question	Relevant criteria
1.	*Uptake.* Do health departments and/or related partners see potential value in the toolkit and make an initial decision to use them?	Utility, feasibility
2.	*Ability to use materials.* Can sites use the guidance and resources provided in the toolkit to implement the data collection protocols to create measurement data?	Feasibility
3.	*Quality of resulting data.* Are the data of high quality?	Validity, reliability
4.	*Perceived benefits.* Do health departments and/or related partners see the toolkit use (e.g., for system improvement, midcourse corrections) as worth the effort?	Utility

**Table 3.** Pilot test sites

Site	Wave	Agency type	Measures used	Incident type
1.	1	State health department	IM measures survey Peer protocol	COVID–19
2.	1	Local health department	IM measures survey Peer protocol	COVID–19
3.	1	Local health department	IM measures survey	COVID–19
4.	1	Local health department	IM measures survey	COVID–19
5.	1	County health department	IM measures survey	COVID–19
6.	1	County health department	IM measures survey	COVID–19
7.	2	State health department	IM measures survey Peer protocol	COVID–19
8.	2	State health department	IM measures survey	COVID–19
9.	2	County emergency management authority	IM measures survey	COVID–19
10.	2	Health care coalition	Peer protocol	Medical surge exercise
11.	2	Local emergency management authority	IM measures survey	COVID–19
12.	2	Local emergency management authority	Peer protocol	Active shooter exercise
13.	2	County fire department	IM measures survey	Wildfire
14.	2	Regional public health coordinator	IM measures survey	COVID–19
15.	2	County public health emergency preparedness authority	IM measures survey	Wildfire
16.	2	County public health emergency preparedness authority*	IM measures survey	Monkeypox
17.	2	Health care coalition*	Peer protocol	Radiation/nuclear exercise
18.	3	County health department	IM measures survey	Flooding
19.	3	County health department	IM measures survey	COVID–19
20.	3	State health department	IM measures survey	Care worker strike (Test 1)
21.	3	State health department*	IM measures survey	Care worker strike (Test 2)
22.	3	Health care coalition*	IM measures survey	Medical surge exercise
23.	3	Indian Health Services hospital**	IM measures survey	Medical surge exercise

* Participated in a previous test.

** Included in previous test but debriefed separately.

**Table 4.** Key findings, by research question and criteria

Research question	Key findings	Relevant criteria
(1) Uptake of tool	• Recruiting agencies to participate in pilot tests was a challenge	Utility, Feasibility
(2) Ability to use tool properly to generate data	• Time spent taking the survey is not particularly burdensome • Respondents are able to answer survey questions, and there is negligible missing data	Feasibility
(3) Ability to use tool to produce high quality data	• Users agree the instrument captures incident management well • There is alignment between how respondents view the management of the incident and the survey scores • The domain summary scores may reliably capture the associated constructs • Survey respondents are willing to provide free-text comments, indicating they took it seriously	Validity, reliability
(4) Users deem tool useful and worthwhile	• Sites believe the tool is useful • Communicability is high but actionability varies	Utility

**Table 5.** Average domain and gestalt scores by site (mean)

Pilot Site	Domain 1	Domain 2	Domain 3	Domain 4	Domain 5	Gestalt A*	Gestalt B*
7 (*n* = 19)	3.83	3.93	3.42	4.03	3.86	3.21	3.68
8 (*n* = 15)	3.48	3.46	2.96	3.87	3.67	3.13	3.57
9 (*n* = 23)	3.96	3.91	3.71	3.93	3.85	3.85	3.67
11 (*n* = 9)	3.86	3.69	3.23	4.08	3.74	3.67	3.78
13 (*n* = 15)	3.90	3.94	3.76	4.09	3.95	3.77	3.54
14 (*n* = 15)	4.05	4.15	4.46	4.48	4.00	4.20	3.40
15a (*n* = 10)	3.85	3.96	3.72	3.84	4.03	4.20	3.70
15b (*n* = 7)	3.85	4.05	4.05	3.99	4.05	4.14	4.00
18 (*n* = 11)	4.16	4.23	3.74	4.32	3.94	4.18	3.18
19 (*n* = 4)	4.19	4.25	4.11	4.25	4.25	3.75	3.75
20 (*n* = 8)	4.50	4.63	4.41	4.55	4.29	4.63	4.75
21 (*n* = 7)	4.47	4.71	4.29	4.59	4.46	4.88	4.38
22 (*n* = 27)	3.92	4.10	4.12	4.17	3.84	4.04	3.96

* Gestalt A: how well the survey captured incident management. Gestalt B: how well the incident was managed.

*Note:* Medians were also computed but differed little from the mean scores.

**Table 6.** Cronbach’s alpha for each domain

Domain	Wave 2 α (*n* = 111)	Wave 3 α (*n* = 58)
1. Situational awareness and information sharing	0.82	0.85
2. Incident action and implementation planning	0.85	0.86
3. Resource management and mobilization	0.84	0.77
4. Coordination and collaboration	0.75	0.85
5. Feedback and continuous quality improvement	0.44	0.45
